# Rats show up to 72 h of significant retention for spatial memory in the radial maze

**DOI:** 10.3758/s13420-024-00633-4

**Published:** 2024-07-24

**Authors:** Chiaki Tanaka, Tohru Taniuchi

**Affiliations:** Graduate School of Socio-Environmental Studies, Kanazawa University, Kakuma, Kanazawa, Ishikawa, 920-1192 Japan

**Keywords:** Rat, Radial maze, Spatial memory, Working memory, Retention

## Abstract

This study explored long-term retention of spatial memory in rats using an eight-arm radial maze. Crystal and Babb (*Learning and motivation*, *39*(4), 278–284, 2008) previously demonstrated that rats could retain spatial memory for up to 25 h in the radial maze. Notably, they found performance improved with 48-h intertrial intervals compared with 24-h intervals. Our study investigated the effects of extending intertrial intervals on long-term retention of spatial memory by reducing the potential for proactive interference. Each trial comprised a learning phase, during which subjects were required to sequentially visit four randomly selected arms, followed by a free-choice test that included all eight arms, conducted after increasing the retention and intertrial intervals. The retention intervals were systematically increased from 1 h to 24, 48, and, ultimately, 72 h, with corresponding intertrial intervals expanding from 24 to 48, 120, and 144 h. Performance significantly surpassed chance levels across all conditions, demonstrating that rats are capable of retaining spatial memory for up to 72 h.

## Introduction

The radial arm maze, developed by Olton and Samuelson (1976), has become a pivotal tool for investigating the memory capacities of rats and other animals. A standard radial arm maze consists of a central platform with eight arms extending radially. Rats can eat food rewards placed at the ends of the arms. In a typical task, where no additional food rewards are given during a trial, the most efficient strategy involves entering each arm only once and avoiding revisits to previously explored arms. The radial arm maze task requires constant updating of memory for visited and unvisited arms, thus requiring the use of working memory. Rats have been reported to perform the task very well (e.g., Olton, 1978; Olton et al., 1977). Such proficient performance in the radial arm maze demonstrates rats’ excellent spatial memory, making it a versatile tool for memory research. It is used not only to assess the basic capacities of working memory, but also to explore complex memory phenomena. These phenomena include memory interference (e.g., Cohen et al., 1994; Roberts & Dale, 1981; Roberts et al., 2017), retrospective and prospective encoding strategies (Cook et al., 1985), episodic-like memory (e.g., Babb & Crystal, 2006), and directed forgetting (Tanaka et al., 2019), as well as neuroscience studies (e.g., Fujihara et al., 2020; Myhrer, 2003).

Some studies have used the radial arm maze to investigate the limits of long-term retention of spatial memory in rats. Beatty and Shavalia (1980) first allowed rats to enter four of the eight arms, and then, after various retention intervals, placed the rats back into the radial maze and conducted a free-choice test with all eight arms open, in which entering any unvisited arm was considered a correct response. The correct response rate did not significantly decrease between retention intervals of 4 min to 4 h, but it declined beyond an 8-h retention, and no significant performance above chance level was observed at a 24-h retention. However, Crystal and Babb (2008) argued that 24 h might not be the limit for retention of spatial memory in rats, rather that the confusion between retention interval and inter-trial interval (ITI) led to around chance performance. In a typical radial arm maze task, one trial is conducted per day, resulting in an ITI of approximately 24 h. Therefore, in Beatty and Shavalia (1980), rats might reset their memory from the learning phase because the 24-h retention interval could be mistaken for an ITI due to a passage of 24 h. This confusion might lead them to perceive the commencement of the test as the beginning of a new trial. In this context, by employing a 48-h ITI, Crystal and Babb (2008) were able to demonstrate that rats exhibited performance significantly above chance levels, even with a 25-h retention interval. This suggests that extending the ITI effectively mitigates the confusion between the retention interval and the start of a new trial, thereby revealing the rats' true capacity for spatial memory retention.

The relationship between the retention interval and ITI is important when considering the effects of proactive interference. In humans, research has shown that increasing the semantic discriminability of memory materials across trials can reduce proactive interference (e.g., Wickens, 1970). Roberts and Dale (1981) argued that the passage of time acts as a critical cue that enables the effective discrimination between memories from previous and current trials, thereby alleviating proactive interference. Cohen et al. (1994) and Wang et al. (2021) investigated proactive interference in rats using a radial maze and reported release from proactive interference if the retention intervals were short (15 s or 1 min) and the ITI was extended to 2 h. This seemingly contradictory result, in which proactive interference no longer occurs with a 2-h ITI despite significant retention of radial maze memory for 4 h (Beatty & Shavalia, 1980), can be explained by the release of proactive interference. This release is not due to forgetting memories from preceding trials, rather to the increased discriminability among memories from different trials, achieved by extending the ITI (Wang et al., 2021). Furthermore, significant performance under a 25-h retention interval in Crystal and Babb (2008) might reflect less proactive interference with a 48-h ITI (Crystal & Babb, 2008) compared with a 24-h ITI (Beatty & Shavalia, 1980).

This study investigated the capacity of rats to retain spatial memory beyond the 25-h mark, hypothesizing that extending the ITIs could mitigate potential proactive interference, thus facilitating long-term memory retention. Similar to the study by Crystal and Babb (2008), a forced-choice/free-choice task was conducted in an eight-arm radial maze. The retention interval was gradually increased from 1 to 24 h to 48 h, while the ITI was adjusted accordingly from 24 to 48 to 120 h. Initially, rats were permitted to freely explore and enter all arms until they collected all the rewards during the free-choice test phase. However, to mitigate concerns that rats might rely solely on immediate memories from the current test phase rather than on long-term memories from the learning phase to choose arms, thereby circumventing the need for long-term memory retention, the protocol was adjusted. In the later phases of the experiment, we increased the reward magnitude given at each arm and limited the number of arm choices available during the test phase to encourage more deliberate decision-making by the rats. Our aim was to compel the rats to make more deliberate selections, optimizing their chances for obtaining food within these constraints and better demonstrating their reliance on long-term spatial memory. Thus, after examining memory retention over 1, 24, and 48 h with unlimited arm choices in the initial test phases, we repeated our investigation with 1-, 24-, and 48-h retention intervals, and included an extension to a 72-h retention interval with an ITI of 144 h, limiting the test phase choices to four arm choices in these new tests.

## Method

### Subjects

We used ten male Sprague Dawley rats obtained from Charles River Japan Inc. The subjects had previously been used in the investigation of proactive interference in a task consisting of a forced-choice learning phase and a free-choice test phase in the radial maze (Wang et al., 2021). After the completion of those experiments, they were subjected to a restricted diet of approximately 18 g of food/day for about 5 months, starting at approximately 320 days of age. In addition to the experimental diet, they received 14 g/day of standard laboratory chow (MM-3, Funabashi Farm Co.). The animals had constant access to water except during the daily experiments. They were housed under a 12:12 light–dark cycle (light period: 8:00 a.m. to 8:00 p.m.). Animal care and experimentation procedures were carried out in compliance with the Kanazawa University Animal Experimentation Regulations, under the approval number AP-214226.

### Apparatus

We used an elevated eight-arm radial maze, at a height of 60 cm above the floor in the experiment room. The maze had a central platform in the shape of a regular octagon, with a 34-cm diameter, extending in eight arms at 45-degree angles. Each arm measured 75 cm in length, 9 cm in width, and was enclosed by gray polyvinyl chloride (PVC) walls 2.5 cm high. The floor of both the central platform and the arms was constructed from plywood and painted with a matte gray finish. At the end of each arm, there was an embedded ceramic food dish with a diameter of 3.5 cm. Transparent acrylic guillotine doors were set at the entrance of each arm, which could be remotely operated to open and close. Illumination was provided by fluorescent lights installed on the ceiling, and the illumination level at the central platform was approximately 400 lx. The experiment room measured 5.0 m in length and 3.5 m in width, and was equipped with various distinctive extra-maze cues. These included a table with two operant boxes, black curtains, a metal shelf, a ceramic sink, and two doors. We used 45-mg food pellets (Bio-serv, F0021) as rewards presented in each food dish. To eliminate any influence of the experimenter's movements on the subjects' behavior, observations and electronic control of the radial maze were conducted from behind a desk, shielded by a semi-transparent black PVC partition screen.

### Procedure

The rats received 1 min of handling for 3 days before the start of reacquisition training for the radial maze task. Habituation to the maze was omitted, as it was anticipated that the rats had already become sufficiently familiar with the maze through their prior experience in Wang et al. (2021). However, reacquisition training was conducted because these rats had not been trained for approximately 5 months following their participation in Wang et al. (2021). During reacquisition training for the radial maze task, each trial consisted of a learning phase, a retention interval, and a testing phase. The learning phase required rats to make sequential forced choices among four specific arms, where ten 45-mg food pellets (450 mg in total) were placed in each food dish. After placing the rat on the central platform, we opened the entrance door to the first arm approximately 5 s later. The rat entered the arm, consumed the food reward, and then returned to the central platform. Upon its return, we opened the door to the next arm while simultaneously closing the door to the arm the rat had just exited. The four arms presented during the learning phase were randomly determined by the experimenter and counterbalanced within and across subjects. The door of each arm visited by the rats was closed one by one during the learning phase, thereby preventing revisits to the arms. The learning phase concluded when the rat had entered all four arms and returned to the central platform. After the learning phase, the experimenter removed the rats to their home cage for a 60-s retention interval. After the retention interval, the testing phase began. In the testing phase, a free-choice task was conducted with all eight arms opened simultaneously. The four arms that the rat had not entered during the learning phase were each baited with ten 45-mg food pellets as rewards. A revisit to arms that had already been entered during the learning or testing phase was considered an incorrect response. The experiment concluded when the rat collected all the pellets placed in the arms and returned to the central platform. Reacquisition training was conducted for 20 days with one trial per day.

After reacquisition training was completed, testing commenced, following procedures similar to those used during reacquisition training. Table 1 provides an outline of the procedures followed during the retention intervals and ITIs, and the number of arm entries allowed during tests across Phases 1–7. Retention intervals were increased from 1 to 24 h and 48 h. The ITIs were extended relative to the retention intervals. For trials with a 1-h retention interval, the ITI was set at 24 h, resulting in one trial per day. For trials with a 24-h retention interval, an ITI of approximately 48 h was used. For trials with a 48-h retention interval, we applied an ITI of approximately 120 h. During trials with retention intervals of 48 h or more, daily feeding was provided during the retention interval. Testing consisted of eight trials for Phase 1 (1-h retention, 24-h ITI), 12 trials for Phase 2 (24-h retention, 48-h ITI), and eight trials for Phase 3 (48-h retention, 120-h ITI). Rats had unlimited access to all the arms so that they could collect food rewards from the four correct arms during the tests across Phases 1–3. All other procedural aspects of Phases 1–3 were identical to those employed during reacquisition training.

**Table 1:** An outline of the procedures followed during the retention interval and inter-trial interval (ITI), the allowed number of arm entries during the tests, and the number of test trials across Phases 1–7. In Phases 1–3, rats had unlimited access to the arms until they collected all the food rewards from the four correct arms during the tests. For Phases 4–7, the number of arm choices during the tests was capped at four

Phase	Retention interval	Inter-trial interval	Allowed number of arm entries during the test	Number of test trials
Phase 1	1 h	24 h	Unlimited	8
Phase 2	24 h	48 h	Unlimited	12
Phase 3	48 h	120 h	Unlimited	8
Phase 4	1 h	24 h	4	4
Phase 5	24 h	48 h	4	4
Phase 6	48 h	120 h	4	4
Phase 7	72 h	144 h	4	4

In contrast with Phases 1–3, the subsequent phases only allowed access to four arms for food rewards. Phase 4 consisted of four trials with a 1-h retention interval and a 24-h ITI. Phase 5 had four trials with a 24-h retention interval and a 48-h ITI. Phase 6 included four trials with a 48-h retention interval and a 120-h ITI. Phase 7 featured four trials with a 72-h retention interval and a 144-h ITI. In Phases 4 through 7, the number of 45-mg food pellets provided at the food dish in each arm was increased from ten to 20. Additionally, during the free-choice test, the number of arms the rats could enter was limited to a maximum of four choices. When a rat returned to the central platform from the fourth arm entered, even if there were unretrieved food rewards remaining in the maze, the trial was terminated. This adjustment was designed to encourage a more cautious arm selection by the rats during the test phase. By increasing the amount of food pellets and imposing limitations on arm entries, we hoped to establish a clear distinction between the amount of food obtainable from correct and incorrect choices. This was expected to enhance the rats' decision-making accuracy during the test. Other aspects of the procedure during Phases 4–7 were identical to those of Phases 1–3.

Fourteen days after completing Phase 7, we conducted a food odor test to assess whether the rats could select arms based on the potential odor cues emanating from the food rewards. We placed 20 45-mg food pellets in four arms randomly selected by the experimenter. After placing the rat on the central platform, we opened all eight arms simultaneously. The doors of each arm visited by the rats were closed one by one to prevent a revisit to the arms. The trial concluded when the rat entered the fourth arm and returned to the central platform, even if unretrieved food rewards remained in the maze. We conducted four test trials with a 144-h ITI. The distribution of baited arms was systematically varied to ensure counterbalancing within and across subjects.

## Results

**Fig. 1:**
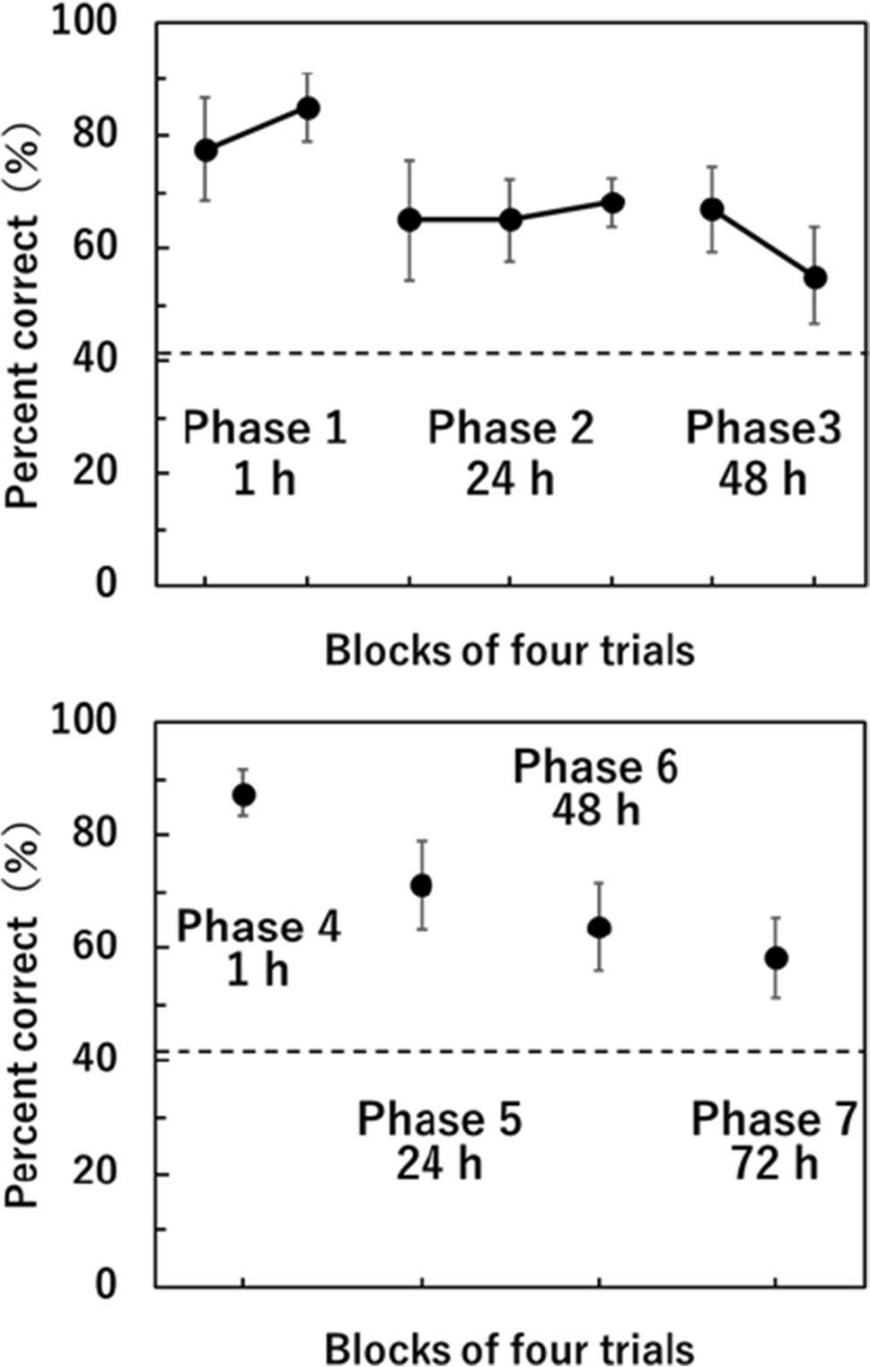
Percentage of correct responses during the first four responses in blocks of four trials. The broken line represents the chance level of 41.38%. Error bars represent 95% confidence intervals of the mean. Rats were allowed unlimited entries into the arms until all rewards were acquired during the free-choice tests in Phases 1–3, while for Phases 4–7, the number of choices in the test was limited to four

Figure 1 shows the mean percentage of correct responses in blocks of four trials. The top panel displays results from Phases 1–3, where the number of choices available during the free-choice test phase was not limited, while the bottom panel shows results from Phases 4–7, in which the number of arm choices during the test phase was restricted to four. Due to illness, one rat was excluded from Phase 7, leading to data for Phase 7 and the later food odor test being based on nine subjects. The percentage of correct responses was determined from the first four responses in each test. Broken lines indicate a chance level of 41.38% (Crystal & Babb, 2008; Roberts et al., 2017; Washizuka & Taniuchi, 2007). For a 1-h retention interval in Phase 1, the 95% confidence intervals (CIs) for the mean were 68.26–86.74 for the first block and 78.96–91.04 for the second block. For a 24-h retention interval in Phase 2, the 95% CIs were 54.42–75.58 for the first block, 57.64–72.36 for the second block, and 63.68–72.57 for the third block. Across all blocks in Phases 1 and 2, the lower limits of the 95% CIs consistently exceeded the chance level, demonstrating significantly better performance for retention intervals of up to 24 h. For Phase 3, with a 48-h retention period, the lower limits of the 95% CIs exceeded the chance level for the first block (59.26–74.49) but not in the second block (46.36–63.64). A paired t-test revealed a significant decrease in performance from Block 1 to Block 2 (*t*(9) = 2.48, *p* = 0.02, *d* = 0.78). These results suggest that rats initially demonstrated reliable performance under 48-h retention intervals, but their performance deteriorated over the course of testing.

In Phases 4–7, with the number of choices in the free-choice test limited to four to potentially induce more cautious arm selection during the test, the 95% CIs were 83.28–91.72 for Phase 4 with a 1-h retention, 63.59–78.91 for Phase 5 with a 24-h retention, 55.92–71.58 for Phase 6 with a 48-h retention, and 51.13–65.54 for Phase 7 with a 72-h retention. In all conditions, the lower limits surpassed the chance level, indicating that rats exhibited reliable test performance, even with 72-h retention intervals.

**Fig. 2:**
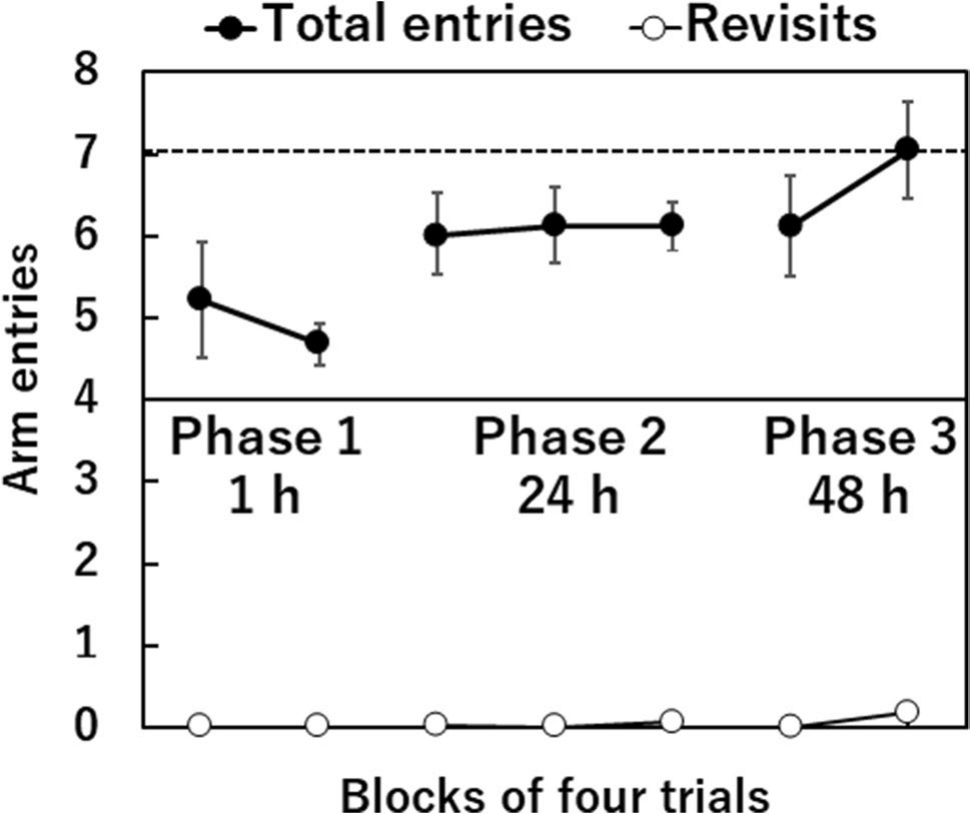
For Phases 1–3, mean number of arm entries required to collect food rewards in four baited arms (solid circle) and revisits (open circle) during the test phase in blocks of four trials. The solid line represents the minimal number of arm entries needed to collect food rewards in four baited arms (4.0). The broken line represents the expected number of arm entries needed to collect food rewards in four baited arms (7.02), assuming the absence of memory use from the learning phase and relying solely on memory during the free-choice test, thus eliminating arm revisits. The expected number of arm entries needed to collect food rewards in four baited arms, assuming neither memory use from the learning phase nor the test phase, would be approximately 16.43. Error bars represent 95% confidence intervals of the mean. During Phases 1–3, rats had unlimited access to enter the arms of the maze until they acquired all food rewards from the four designated arms in the free-choice test phase

Figure 2 depicts the average number of arm entries and revisits to previously entered arms required to retrieve all food rewards from the four correct arms, presented across Phases 1–3 in blocks of four trials. The horizontal broken line represents the expected value 7.02 of the number of arm entries when rats rely solely on short-term memory retention to avoid revisiting arms during the current test phase, without utilizing memories from the learning phase (random choice without replacements). If rats did not utilize both short-term memory retention during the current test phase and memories from the learning phase (random choice with replacements), the expected value would be 16.43 (based on the Monte Carlo method over 100,000 iterations). CIs in Fig. 2 demonstrate that rats performed significantly better than the expected chance of 7.02 through the first block of Phase 3 (48-h retention), suggesting rats could retain memories of arms from the learning phase for up to 48 h. However, their performance deteriorated in the second block of Phase 3 (48-h retention), with the 95% CI of 6.47–7.63 not significantly differing from the chance level of 7.02, which assumes short-term memory retention during the current test phase. Nevertheless, performance remained significantly better than the chance level of 16.43, which assumes that neither short-term memory retention during the current test phase nor memories from the learning phase were utilized. These results suggest that rats initially retain memories from the learning phase for 48 h, but gradually abandon retention of those memories over time. Considering the number of revisits to arms was almost zero in the second block of Phase 3, the results suggest that rats may have abandoned retention of memories from the learning phase for 48 h. Instead, they appeared to rely solely on short-term memory retention for arm entries, avoiding revisits to arms that were not baited.

After completing Phase 7, we investigated whether the rats were capable of identifying the baited arms by detecting the odor of food rewards, which had been placed in the dishes located at the ends of the arms. Specifically, we evaluated if the rats could identify the four baited arms solely by the odor of food rewards, achieving an accuracy greater than the 50% chance level within their initial four choices from the eight arms. With 20 45-mg food pellets distributed across four randomly selected arms of the radial maze, the observed average selection rate for these baited arms in the initial four choices was 47.92% (95% CI: 37.50–62.50). The observed rate of arm selections was not significantly higher than the chance level of 50%, indicating that the rats' arm selections were not significantly influenced by the odor of food rewards.

We also explored the possibility of scent marking, whereby rats might have marked arms with their scent during the learning phase and avoided these in the test phase throughout Phases 1–7. If this behavior occurred, it would likely persist during the food odor test conducted after Phase 7. To investigate this, we analyzed the selection rate of arms entered in the immediately preceding trial during the four trials of the food odor test. It was expected that rats would significantly avoid the four arms they had entered in the immediately preceding trial conducted approximately 72 h ago if they had learned to avoid the arms based on their own scent marking by Phase 7 (72-h retention). For example, it was expected that if rats used scent marking to identify previously visited arms, they would avoid those arms in the second, third, and fourth trials of the food odor test, corresponding to their initial visits in the first, second, and third trials, respectively. However, the selection rate of these four arms that had been entered in the immediately preceding trial was 52.78% (95% CI: 42.16–63.40), which was not significantly lower than the chance level of 50%. We found a similar selection rate of 49.07% (95% CI: 30.71–67.44) for the arms where food rewards had been consumed in the immediately preceding trials. These findings strongly suggest that the rats' performance during Phases 1–7 was not dependent on their own scent marking on the arms.

## Discussion

In this study, we examined rats' spatial memory retention beyond the 25-h retention interval reported by Crystal and Babb (2008) by adjusting the ITIs according to the length of the retention intervals. This adjustment aimed to minimize potential proactive interference from preceding trials. Our findings revealed that rats achieved significantly higher test performance than the chance level, even after a retention interval of 72 h. This indicates that rats are capable of retaining memory of the radial maze for up to 72 h. Crystal and Babb (2008) observed a significant improvement in performance when extending the intertrial interval (ITI) from 24 to 48 h, measured after a 25-h retention interval. This underscores the need to differentiate between retention intervals and ITIs to prevent potential confusion. Moreover, extending ITIs is pivotal in reducing proactive interference, where memories from earlier trials negatively impact performance in later trials, a factor especially critical in studies of spatial memory retention (Roberts & Dale, 1981; Roberts et al., 2017). Studies have shown that release from proactive interference occurs when ITIs are sufficiently long, such as 2 h (e.g., Cohen et al., 1994; Wang et al., 2021). The release from proactive interference could be attributed to the enhanced discriminability between memories of preceding and current trials, facilitated by longer ITIs (Roberts & Dale, 1981; Roberts et al., 2017). In our study, ITIs were systematically extended in relation to the retention intervals, up to a 144-h ITI for a 72-h retention interval. This approach, aligning longer ITIs with extended retention periods, may have contributed to the observed evidence of long-term retention of spatial memory of up to 72 h. Further extension of the ITIs could potentially facilitate the retention of spatial memory in rats over retention intervals longer than 72 h.

By extending the ITIs, not only can proactive interference be reduced, it is also possible that this constitutes a spaced training condition. Traditionally, spaced training is superior to massed training in various learning tasks in humans and non-human animals (e.g., Smolen et al., 2016, for a review). Some cognitive theories have explained the superiority of spaced training by suggesting that it leads to the encoding of a memory of the task in multiple contexts, the effective retrieval and reactivation of a memory formed by the preceding trial, or the strong consolidation of a memory. It is not surprising that spaced training might contribute to acquisition in the radial arm maze task, especially given the unusually long retention intervals used in the present study. However, determining the effectiveness of spaced and massed training on the acquisition of the radial arm maze task has never been examined. Comparing spaced versus massed training could be an interesting research topic, especially in a difficult task such as long-term memory retention in the radial maze.

During the initial Phases 1–3, rats were permitted unlimited entries into the arms until they had acquired all food rewards from the four designated correct arms during the free-choice tests. However, for the later Phases 4–7, we limited the number of choices in the test to four. This adjustment was made because allowing unlimited arm entries during the free-choice test of Phases 1–3 might lead rats to abandon retaining and using memories from the learning phase, opting instead to avoid re-entering arms during the current test phase using short-term working memory, a strategy that demands less cognitive effort, rather than focusing on long-term memory retention. Indeed, in Phase 3, under the 48-h retention condition, the lower limit of the 95% CI for the second block dropped to near the chance level, while the mean number of revisits during the test phase remained almost zero. By limiting the choices to four, we observed that performance consistently exceeded the chance level, not only during the 48-h retention interval in Phase 6 but also throughout the 72-h retention interval in Phase 7. This suggests that limiting the number of choices in the test phase prompted the rats to develop more cautious selection criteria for the arms, thereby improving performance. However, beginning in Phase 4, we not only limited the number of arms the rats could enter during the test phase but also increased the food pellets in the dish. This was done to create a clearer distinction between the rewards for correct and incorrect choices, aiming to improve the rats' decision-making accuracy during the tests. Therefore, to be precise, we cannot separately evaluate the effects of limiting the number of arms the rats could enter during the test phase and of increasing the food reward amount. To rigorously investigate long-term retention of spatial memory in rats, it is crucial to individually assess the effects of these treatments and their interactions.

Studies have shown that performance improvements in the radial maze can result from modifications to the arms. Brown and Lesniak-Karpiak (1993) found by using white floors, which rats may find aversive, or incorporating an incline to increase the response load, these treatments may contribute to improvements in performance in the radial maze by influencing the criteria for arm selection by rats. Besides limiting the number of choices or increasing the food rewards in this study, introducing additional challenges, such as arms with white floors or incorporating inclines, could potentially enhance performance.

This study demonstrated performance significantly above chance level, even after retention intervals of 72 h. However, it is possible that strategies other than long-term memory retention might explain this enhanced performance. For instance, rats might have chosen arms during the free-choice test based on the odor of food pellets placed in the dishes at the end of the arms. In the test phase, food pellets were only placed in the arms that had not been entered during the learning phase. Therefore, it was possible that rats selected arms based on the odor of food pellets during the test phase. To test this possibility, we assessed whether the rats could detect the baited arms through the odor of food rewards, which would result in a selection performance exceeding the chance level. However, the selection rates for the baited arms did not surpass the chance level, suggesting that the odor of food rewards did not significantly influence the rats' choices of arms.

Alternatively, rats might have performed the task by marking the arms they entered during the learning phase with their own scent and avoiding those marked arms during the test phase. Some studies have shown that rats possess an excellent sense of smell, allowing them to discriminate between their own and other individuals' odor traces (e.g., Wallace et al., 2002). However, it is established that rats do not depend on potential olfactory cues for navigation in the radial arm maze; rather, they differentiate between previously entered and unentered arms using non-olfactory, distal visual cues from the surroundings of the maze (e.g., Mazmanian & Roberts, 1983; Olton & Collison, 1979; Suzuki et al., 1980).

In this study, the maze was wiped clean with a wet cloth after each rat's trial (during both learning and test phases) and with a cloth soaked in disinfectant alcohol at the end of each experimental session. Furthermore, during the intertrial or retention intervals of 24 h or more, another experiment using the same radial maze was conducted, and similar cleaning procedures were followed. Crystal and Babb (2008), who demonstrated the retention of memory in a radial maze for 25 h, employed procedures that are similar to those used in the present study. It appears impossible for rats, under these procedures, to discriminate between visited and unvisited arms based on possible scent marking after a few days.

We also examined the potential use of scent marking, where rats might have performed the task by marking the arms they entered during the learning phase with their own scent and subsequently avoiding these marked arms during the test phase. If rats were capable of distinguishing between previously visited and unvisited arms, based on the scent of their own markings on the arms, one would expect them to avoid the four arms they entered in the immediately preceding trials during subsequent trials. However, the selection rate of those four arms, entered in the immediately preceding trials during the food odor test, did not differ from chance levels. We found similar results for the selection rate of the arms in which they had consumed food rewards in the immediately preceding trials. Therefore, it is highly unlikely that rats in this study performed the radial maze task based on lingering olfactory cues on the arms. However, for a more rigorous demonstration of long-term spatial memory retention in rats' performance in the radial maze, it might be necessary to employ methods that strictly eliminate the potential effects of olfactory cues. One approach could involve rotating the arms of the radial maze between the learning and test phases, as suggested by Olton and Collison (1979).

The present study demonstrated significant retention of spatial memory for up to 72 h in the radial arm maze task in rats. However, other studies focusing on source memory have reported longer memory retention (e.g., Crystal & Alford, 2014; Crystal & Smith, 2014; Crystal et al., 2013). Source memory refers to memories about the conditions under which a memory was acquired, including perceptual, contextual, temporal, affective, and other features that were present when the memory was formed (Crystal et al., 2013). For example, Crystal et al. (2013) investigated source memory in rats by examining whether they could retain information about the source of their memory of retrieving the preferred food, chocolate: whether they retrieved it by entering an arm of the radial maze themselves or by being placed at the end of the arm by the experimenter. During the test phase conducted after the retention interval, the preferred food was again placed in the arm where the rats had entered by themselves, but not in the arm where they had been placed by the experimenter. In this two-choice discrimination, the rats showed significantly above-chance performance even after a 7-day retention interval. In contrast, the performance of spatial memory assessed by the typical win/shift task with six arms deteriorated in about 2 days. While the focus in radial maze tasks has been on the retention of spatial memory, rats seem to retain and utilize the information of “how” they found the food, and such source memory might be more enduring than spatial memory. However, source memory of rats in radial maze performance has mainly been investigated using a two-arm choice task mixed into a typical spatial memory task with six arms (e.g., Crystal et al., 2013). To compare retention between spatial memory and source memory directly, it might be interesting to conduct experiments in the radial maze using the same number of arms for learning as in typical spatial memory tasks. For example, four arms could be used for learning each source memory: either entering by themselves or being placed there by the experimenter. Further investigation is needed to elucidate characteristics, including persistence of both spatial memory and source memory.

There are some non-typical features in the procedure of this study: the subject rats' previous experience, the gradual increase of retention intervals, and the use of larger rewards given at each arm. As described in the *Method* section, the subjects had previously been used in an experimental investigation of proactive interference in the radial maze (Wang et al., 2021). Additionally, we trained the rats with gradually increasing retention intervals of 1, 24, 48, and then 72 h. It is not clear whether such extensive training experience or the gradual increase in retention intervals contributes to the long-term retention of spatial memory for up to 72 h. Moreover, we used a larger food reward of ten pellets (Phases 1–3) or 20 pellets (Phases 4–7) at each arm compared to the typical one pellet used in standard radial maze procedures (e.g., Crystal & Babb, 2008). The use of larger rewards might contribute to successful performance under unusually long retention intervals in this study. Salvetti et al. (2014) reported that rats showed significant performance in recognizing the spatial position where they ate three food pellets 24 h earlier, but only chance performance if they ate just one food pellet at the position. Clearly, the effects of training levels, training steps, and reward magnitude on long-term retention in the radial maze need to be examined.

Considering that rats at 24 months are regarded as aged (e.g., Kadar et al., 1990), another interesting feature of this study is significant retention of spatial memory for 72 h in Phase 7 by rats that were approximately 19 months old at the start of this phase. Studies have shown that memory declines with age in rats in radial maze performance. For instance, Kadar et al. (1990) compared the performance of young (3-month-old), adult (12-month-old), middle-aged (17-month-old), and aged (24-month-old) rats using a task involving retrieving food placed in all arms of the radial maze. This task primarily required short-term working memory for remembering the arms already visited in a trial, rather than long-term retention of visited arms. The young group exhibited significantly higher correct response rates compared to the other groups. Moreover, the performance of the adult and middle-aged groups was similar, whereas the aged group showed poorer performance compared to these groups. Therefore, although not as pronounced as in aged rats, there are reports of decreased performance in rats aged approximately 12–17 months compared to younger individuals. While there were no control conditions involving young rats, the findings of this study seem remarkable in demonstrating significant retention of spatial memory in 19-month-old middle-aged rats for as extended a period as 72 h.

The concept of cognitive reserve suggests that certain individuals maintain good cognitive performance despite neural damage or age-related declines in brain function (e.g., Ebina et al., 2023; Stern, 2009). Cognitive reserve in aged people is linked to prior education and occupation entailing engagement in intellectual activities (Kramer et al., 2004). In this study, the subject rats were previously used in proactive interference tasks in the radial maze, as detailed in Wang et al. (2021), and received ongoing training. Their extensive exposure to spatial memory tasks from an early age, essentially engaging them in "intellectual activities," might have influenced their performance in this study. However, it is uncertain whether the 72-h memory retention observed in 19-month-old rats is a direct outcome of their prior spatial memory task experiences. It is essential to investigate the impact of such previous, continuous cognitive task engagement on the spatial memory capabilities of aged rats, particularly in the context of cognitive reserve. Furthermore, since memory retention was possible for 72 h in middle-aged rats, it may be possible for even longer retention in young rats that might have superior memory abilities. It would be interesting to examine the limits of memory retention in the radial maze with young rats compared with the effects of extensive experience in cognitive activities over a long period in aged rats.

## Data Availability

The datasets are available from the corresponding author on reasonable request.
